# Supplementary material for: Mechanisms governing the pioneering and redistribution capabilities of the non-classical pioneer PU.1
Source: Nat Commun. 2020 Jan 21;11:402. doi: 10.1038/s41467-019-13960-2 (PMC6972792; doi:10.1038/s41467-019-13960-2)
Supplement: Supplementary file 7 — Source data [file 41467_2019_13960_MOESM7_ESM.zip › Source_Data/FigureS3/FigureS3J_MotifScanOutput/homerResults/motif7.similar.html]

motif7

## Information for motif7

G
C
A
T
C
A
T
G
C
T
A
G
T
G
C
A
G
T
A
C
G
C
A
T
A
C
T
G
G
A
C
T
  
Reverse Opposite:  

C
T
G
A
T
G
A
C
C
G
T
A
C
A
T
G
A
C
G
T
G
A
T
C
G
T
A
C
C
G
T
A
  

|  |  |
| --- | --- |
| p-value: | 1e-154 |
| log p-value: | -3.561e+02 |
| Information Content per bp: | 1.625 |
| Number of Target Sequences with motif | 5781.0 |
| Percentage of Target Sequences with motif | 43.81% |
| Number of Background Sequences with motif | 11269.1 |
| Percentage of Background Sequences with motif | 30.87% |
| Average Position of motif in Targets | 408.8 +/- 386.9bp |
| Average Position of motif in Background | 321.0 +/- 205.0bp |
| Strand Bias (log2 ratio + to - strand density) | 0.0 |
| Multiplicity (# of sites on avg that occur together) | 1.36 |
| Motif File: | file (matrix) reverse opposite |

### Similar de novo motifs found

|  |  |  |  |  |  |  |  |
| --- | --- | --- | --- | --- | --- | --- | --- |
| Rank | Match Score | Redundant Motif | P-value | log P-value | % of Targets | % of Background | Motif file |
| 1 | 0.862 | C T G A T G A C C G T A A C G T C G A T A T G C A G T C C G T A C T A G | 1e-141 | -325.945082 | 24.45% | 14.46% | motif file (matrix) |
| 2 | 0.892 | A T G C C G A T A G T C C G A T A C T G T C A G G T C A G T C A C G A T A C T G G A C T | 1e-140 | -323.203753 | 25.94% | 15.71% | motif file (matrix) |
| 3 | 0.801 | T A C G G T A C G C A T A C T G A C T G C G T A C G T A A C G T A C T G A G T C | 1e-129 | -297.500195 | 20.76% | 11.88% | motif file (matrix) |
| 4 | 0.859 | C A T G G C T A G T A C C G A T C A T G T C A G G C T A T C G A C G A T A C T G A G C T C A G T C A G T | 1e-128 | -295.213598 | 18.46% | 10.11% | motif file (matrix) |
| 5 | 0.863 | C A G T C T G A T G A C C G T A A C G T A C G T A T G C A G T C C G T A C A T G G A C T T A G C | 1e-123 | -284.855246 | 24.84% | 15.37% | motif file (matrix) |
| 6 | 0.831 | C T G A G T A C C G T A A C G T A C G T A G T C A G T C | 1e-111 | -256.927819 | 24.26% | 15.31% | motif file (matrix) |
